# The Relationship between Insecure Attachment to Depression: Mediating Role of Sleep and Cognitive Reappraisal

**DOI:** 10.1155/2020/1931737

**Published:** 2020-04-14

**Authors:** Yige Liu, Hongfan Li, Xiayue Xu, Yukun Li, Zhutao Wang, Huan Zhu, Xia Zhang, Shan Jiang, Naling Li, Simeng Gu, Fushun Wang, Jason H. Huang

**Affiliations:** ^1^Depatment of Psychology, School of Medicine, Jiangsu University, Zhenjiang 212013, China; ^2^Institute of Brain and Psychological Sciences, Sichuan Normal University, Chengdu 610060, China; ^3^Department of Neurosurgery, Baylor Scott & White Health, Temple, TX 76502, USA; ^4^Department of Surgery, Texas A&M University College of Medicine, Temple, TX 76502, USA

## Abstract

Previously, we have shown that neuromodulators are important factors in stress-induced emotional disorders, such as depression, for example, serotonin is the major substance for depression. Many psychological studies have proved that depression is due to insecure attachment. In addition, sleep is a major symptom of depression. Furthermore, serotonin is the substrate for both sleep and depression. To explore the role of sleep in the relationships between insecure attachment and depression, we investigated 755 college students with Close Relationship Inventory, Emotion Regulation Questionnaire, Self-rated Depression Scale, and Pittsburgh Sleep Quality Index. The results showed that (1) insecure attachment positively predicted poor sleep quality; (2) sleep quality partially affected depression, possibly due the same stress neuromodulators such as norepinephrine and cortisol; and (3) cognitive reappraisal moderated the mediating path leading from attachment anxiety to poor sleep quality. These findings highlight the moderating role of cognitive reappraisal in the effects of attachment anxiety on sleep quality and finally on depression. In conclusion, sleep quality links attachment anxiety and emotional disorders.

## 1. Introduction

Emotion disorders, such as depression is the leading cause of disability worldwide [1]. However, there is no efficient treatment for this disease, for the underlying neural mechanism of emotions is unclear [2]. The most widely accepted theory about affective disorders is the monoamine theory, which suggests that monoamines, including norepinephrine, dopamine, and serotonin (5-HT) are the major reasons for emotion disorders [3], and the first line of treatment for depression also targets these monoamines [4]. However, there are still many controversies about monoamine neurotransmitters affecting the emotion processes, for example, it is well known that 5-HT is a major neurotransmitter that is involved in depression; and sleep is also modulated by 5-HT in raphe nuclei, but how sleep affects depression is not clear. Our previous studies suggested that monoamine is related to emotional arousal [5], which might affect sleep, and poor sleep might in turn causes emotional depression [6].

Sleep disorder has become a health problem that affects almost all the people around the world. The 2017 Nobel Prize in biology and medicine has been awarded to three American scientists, Michael, Jeffrey, and Michael, for their work in studying the neural mechanisms by which the body clock works, highlighting the importance of sleep. Many papers are still reporting the mechanisms and functions of sleep, for example, it is found that sleep helps metabolite clearance [7]. In addition, it is found that sleep can also affect the glymphatic system to clear the big molecules in the brain [8], and it is reported that glymphatic system might induce depression in Alzheimer's disease [9]. Our previous studies also reported that sleep is an important time for the recuperation and rejuvenation of the brain [10]. In all, sleep can induce many kinds of neurological and psychological diseases, such as emotion disorders, substance use, low academic performance, and physical dysfunctions [11]. Unfortunately, the underlying neural mechanism of sleep problems is not clear yet, sleep-related psychological factors have got little empirical results yet.

Some studies found that sleep quality may be affected by one dispositional factor: insecure attachment [12]. Attachment theory proposed by Bowlby suggested that an individual's personality development and emotional disorders are associated with maternal and infant attachment, and the destruction of attachment bonds in childhood can lead to a lifelong emotional disorders, manifested as a sudden onset of depression or anxiety [13]. For nearly half a century, many investigations have probed into the mechanisms about attachment theory, including neural changes underlying the attachment [14]. Many studies have found that insecure attachment is related to many kinds of mental disorders, such as depression [15], and many studies also showed that attachment is related to stress responses, for example, maternal deprivation can enhance activity of the hypothalamic-pituitary-adrenal axis [16], thus release of cortisol, which consequently leads to monoamine dysfunction and thus sleep problems and depression [17]. Overall, unsafe mother-infant attachment can lead to sleep problems in infants and young children. Unfortunately, studies on sleep in the relationship from attachment to depression is relatively lacking.

Attachment theory holds that early attachment relationships influence the brain development, and also epigenetics [18]. These changes, in turn, act as explanatory filters to influence and guide the individual's future cognitive and emotional processing (attention, memory, anticipation, attribution, etc.), which further shapes the individual's attachment development and emotional health in adulthood. Thus, we speculate that, similar to the adverse effects of maternal and infant insecure attachment on individual sleep, adult insecure attachment may also have an adverse effect on the sleep quality of adult individuals and thus emotional disorders. Even though some studies have also suggested that adults with insecure attachment reported poorer sleep quality than those with secure attachments [19]. However, the underlying relationship among them is not clear, for example, which dimensions of attachment predict sleep disturbance, avoidance, or anxiety or both? Or what factors can possibly be the pathways? Here, we try to probe in the causal relationship among them.

### 1.1. Mediating Effect of Sleep

**Figure 1:**
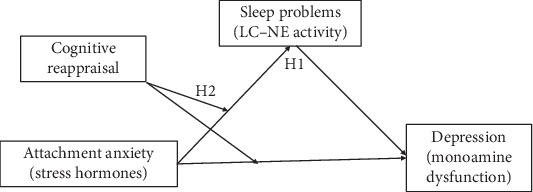
The hypothetical model.

Many reports suggested that insecure attachment is closely related to emotional disorders, such as depression, because they are all influenced by the HPA axis and monoamine systems. In addition, insecure attachment can induce sympathetic nervous system and norepinephrine release, which affect sleep qualities. Sleep problems in turn will affect the physical and mental states, including emotional disorders. Thus, we hypothesize that adulthood insecure attachment positively predicts an individual's sleep quality. Sleep problem has been proved to impair college students' mental and physical health, and sleep disorders can cause depression-like behaviors [20]. Thus, we hypothesize that sleep problem mediates the relationship between adulthood insecure attachment and depression (H1) (Figure 1).

### 1.2. Mediating Effects of Cognitive Reappraisal

Cognitive reappraisal is an emotional regulation strategy that reduces emotional response by changing the re-recognition of emotional events. Cognitive reappraisal is not only a well-adapted response but also a positive adaptive ability. People who adopt positive strategy can actively change their perception of the personal meaning of emotional events or their own understanding of emotional events [21]. Previous studies show that cognitive reassessment plays an important role in improving psychological adaptability, protecting established interpersonal relationships, and promoting mental health [22]. Our previous studies suggested that reappraisal is important for good sleep and positive emotions [23]. Early cognitive behavioral therapy also aims to alleviate negative emotions and eliminate bad behaviors by adjusting and changing the distorted cognitive model of the interviewee and improving cognitive reappraisal ability.

According to the reverse regulation theory, cognitive reappraisal can help people correctly evaluate the information in the current situation and prevent impulsive behaviors [24]; in addition, it can also reduce HPA axis-releasing cortisol, reduce emotional arousal, and induce good sleep. In other words, cognitive reappraisal can reduce stress hormone release and promote individuals' adaptation to emotional factors, improve response quality, and help individuals maintain emotional balance. Risk buffer model points out that protective factors can buffer or weaken the adverse effects of risk factors [25]. Studies have confirmed that cognitive reappraisal can reduce norepinephrine-induced emotional arousal, and thus reduce the risk effect of negative coping style. Thus, we predict that cognitive reappraisal affects the adult insecure attachment-induced depression. Therefore, we hypothesize that the mediating effects of sleep quality would be weaker in individuals with high cognitive reappraisal than those with low cognitive reappraisal level (H2).

To sum, we intend to investigate the relationships among adulthood insecure attachment and depression as well as its mediating and moderating mechanisms. Based on previous theories and studies, we hypothesize that sleep problem would mediate the effects of insecure attachment on depression, and the mediating effect would be regulated by the level of cognitive reappraisal. The hypothetical model is shown in Figure 1.

## 2. Methods

### 2.1. Participants

867 college students in Jiangsu province participated in our survey. All the procedures are done according to the ethic assessment and were approved by the Human Research Ethics Committee of Jiangsu University. The participants were informed and gave a written consent. 53 surveys were incomplete, and an additional 59 participants reach the excluding criterion (with obvious psychiatric diseases, such as depression). In all, 755 (87.08%) usable surveys were obtained from the students, which included 298 (39.47%) male and 457 (60.53%) female, averaged age is 19.8 ± 2.3 years (SD = 2.41, range = 17-25 years). 262 (34.70%) participants were freshmen, 299 (39.60%) were sophomore, and 193 (25.70%) were juniors or above. No significant difference in grade distribution between the genders of the participants was found (*χ*^2^(3) = 1.02, *p* = 0.80).

### 2.2. Materials

#### 2.2.1. Attachment

Adulthood insecure attachment was investigated with the Chinese version of the Experience in Close Relationship Inventory (ECR), which includes 36 items and two dimensions: attachment anxiety and attachment avoidance. Each item is scored from 1 to 7 (1 means “not at all consistent” and 7 means “very consistent”). After reversing the score of some items, the score of all the questions was calculated. A higher score indicates less attachment security. The Chinese version of the scale has got good validity and reliability and is wildly used among Chinese mainland students. And we got the Cronbach's alpha to be 0.71 for the avoidance dimension subscale and 0.88 for the anxiety dimension subscale.

#### 2.2.2. Self-Rated Depression Scale (SDS)

Self-Rating Depression Scale (SDS), compiled by Zung (1965), was used to measure the students' depression level. There are 20 items in this scale, and a scale 0-4 was scored for each item. A higher score means a higher degree of depression. This scale is easy to operate, and the score is not affected by age, gender, economic status, and other factors. It is one of the commonly used self-measuring scales for depression [26]. In this study, the confirmatory factor analysis results of the questionnaire showed good structural validity, and the fitting indicators were as follows: *x*^2^ = 1.903, *df* = 1, TLI = 0.950, RMSEA = 0.077, CFI = 0.992, GFI = 0.994, and the Cronbach's *α* is 0.762.

#### 2.2.3. Cognitive Reappraisal

Cognitive reappraisal was measured by the Chinese version of the Emotion Regulation Questionnaire. The scale includes 6 items, with each item scoring from 1 to 7 (1 means “strongly disagree” and 7 means “strongly agree”). A higher score indicates more frequently emotional regulation strategies. The Chinese version of the scale has been wildly used in Chinese college students' samples and shows great reliability and validity. Cronbach's alpha of the ERQ subscale was 0.78.

#### 2.2.4. Sleep Quality

Pittsburgh Sleep Quality Index (PSQI) was used to measure the sleep quality. The questionnaire includes 7 factors and 19 items. The 7 factors include sleep latency, sleep duration, sleep disturbance, sleep efficiency, daytime dysfunction due to sleepiness, as well as sleep medication use, and overall sleep quality. Each item was scored with 0-3, with high scores indicating worse sleep. This questionnaire is widely used in China and shows good validity and reliability. 0.87 was the Cronbach's alpha for this study.

#### 2.2.5. Procedures

Paper-and-pencil-based questionnaires were used in this study, and the investigation was done in 30-60 student classes. The investigation was done during the middle semester and excluded stressful events such as examinations. The test lasted for about 30 minutes. SPSS20.0 and SPSS macro PROCESS were used for the statistics.

## 3. Results

### 3.1. Preliminary Analyses

We first checked the normality, using the asymptotically distribution-free procedure, and the results showed that the data are univariate normality. Then, we evaluated the common method variance using the Harman's single-factor test and found that the maximum component explained only 19.36% of total variance, which suggested that no single factor can explain majority of variance.

The means and standard deviations, as well as correlations among all variables, are shown in Table 1. The mean PSQI score was 6.37 ± 2.72 among 755 students, and 223 students (97 males, 126 females) showed sleep disturbance (PSQI scores ≥ 8), with no difference between male and female (*t* = 0.61, *p* = 0.54). The average score for depression was 3.83 ± 0.59. Correlation analysis showed that insecure attachment is positively related with depression (*R*_2_ = 0.33), and also with sleep quality (*R*_2_ = 023). Depression is also correlated with sleep quality (*R*_2_ = 027). However, reappraisal is negatively correlated with attachment anxiety.

### 3.2. Mediation Analyses

The indirect effect of sleep on the relationship between insecure attachment and depression was examined using the SPSS 20.0 PROCESS procedure. The results show that insecure attachment positively predicted depression (*β* = 0.22, *p* < 0.001); and insecure attachment also affected sleep quality (Table 2, *β* = 0.51, *p* < 0.001). If both attachment anxiety and sleep quality are taken as predictors, their effects on depression are significant (*β* = 0.32, *p* < 0.01 and *β* = 0.84, *p* < 0.001).

In order to further test the indirect effects between them, we created 1,000 bootstrap samples, and the results are shown in Table 2. The indirect effect for sleep quality was statistically significant (indirect effect = 0.18, 95%CI = 0.11-0.27), and the ratio of indirect to total effect was 36.56%, which means sleep quality serves a partial mediating function in the relation between insecure attachment and depression. Later, we tested the indirect effects of reappraisal on the attachment avoidance and depression, and the result indicated that sleep did not serve as mediating role (95%CI = 0.00-0.11).

### 3.3. Moderation Analysis

Then, we used SPSS 20.0 PROCESS analysis to test the moderation effects of reappraisal on the links between attachment and depression through sleep quality. It was found that attachment anxiety positively predicts sleep quality (Table 3, *β* = 0.21, *p* < 0.001), and sleep quality positively predicts depression (*β* = 0.80, *p* < 0.001). In addition, the standardized regression coefficient of “attachment anxiety × cognitive reappraisal” negatively predicts sleep quality (*β* = −0.08, *p* < 0.001) and cannot predict depression, which implies that the indirect effect from attachment anxiety to depression is moderated by the level of cognitive reappraisal, and cognitive reappraisal has no moderating effect on the direct effect of attachment anxiety on depression.

**Figure 2:**
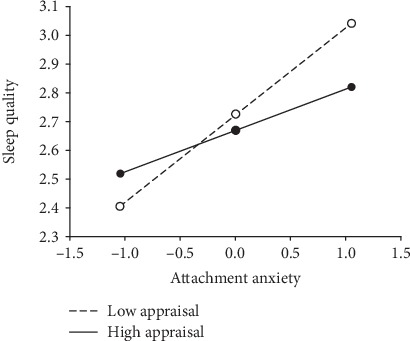
Simple slope analyses of the moderating effect of cognitive reappraisal.

We further investigated with moderation effects using a simple slope analysis and found that there is a stronger positive relationship between attachment anxiety and depression in individuals with lower levels of cognitive reappraisals (Figure 2 and Table 4). In contrast, individuals with higher levels of cognitive reappraisal tend to show a weaker positive relationship between attachment anxiety and depression.

**Figure 3:**
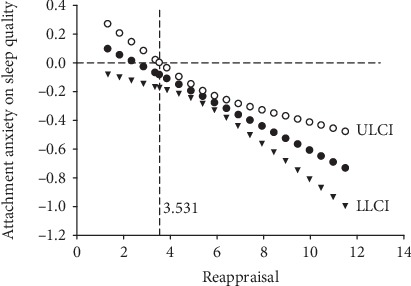
Effects of attachment anxiety on sleep quality moderated by cognitive reappraisal (*LLCI* and *ULCI* represent the lower and the upper limitations of 95% bootstrap confidence interval, respectively).

To explore the boundary value of the moderating effect, we used the Jonson-Neyman technique (J-N technique) [25]. And the data showed that attachment anxiety affects depression less dramatically with the increasing cognitive reappraisal and became statistically significant for participants whose scores were over 3.53, which contained 94.70% participants in this study (Figure 3). The results indicate that for most individuals, the moderating effect of reappraisal is significant. Of note, 95% confidence interval is required in the J-N technique, which suggests that “0” means there is no association between attachment anxiety and sleep quality (Figure 3).

## 4. Discussion

Tons of studies have suggested that insecure attachment are related to stress-induced depression [27–28]; however, the underlying neural mechanisms are not clear [29]. We proposed that the neuromodulators are the major substrate for emotions, also for sleeps and attachments [30]. Thus, we propose that insecure attachment positively predicts depression and poor sleep quality mediates the process. Insecure attachment positively predicts poor sleep quality, and poor sleep quality can induce bad emotions, such as depression. Consistently, our previous work showed that depressive people showed characteristic EEG recordings [31]. The data in this study which are collected from 755 college students in the Jiangsu, province of China, support our model.

First, the current study illustrates the relationship among adult attachment anxiety and sleep quality as well as depression. Similar to the attachment in kids, insecure attachment in adults is also related to emotional disorders, such as depression. The results showed that insecure attachment (represented by high level of attachment avoidance and/or high level of attachment anxiety) reported poorer sleep quality than those with secure attachment, which means self-reported sleep problems are positively associated with insecure attachment. This finding was consistent with previous studies with adult samples [32]. According to attachment theory, individuals with insecure attachment often meet social relationship problems. Individuals with attachment anxiety tend to be stressful and have HPA axis activated and tend to experience sleep disorders [33]. In addition, individuals with attachment avoidance usually have the amygdala activated to have fearful emotions, and thus downplay their close relationships, and they are not successful in using good social support and do experience emotional arousal [34].

Secondly, the data also show the effect that attachment anxiety positively affects poor sleep quality which is moderated by an individual's cognitive reappraisal level. Or put it another way, attachment anxiety affects sleep quality less in individuals with higher cognitive reappraisal compared with those individuals with low cognitive reappraisals [35–36]. Our data from the J-N analysis confirmed this point that reappraisal plays an important role between attachment anxiety and sleep quality. We suppose that both forms of insecure attachment can predict sleep problem by this mechanism, but the results do not support this. Consistently, the attachment theory proposed that avoidant individuals would not trust others and believe they cannot depend on others either [37].

Finally, the results in this study would shed some lights on the mechanism about how insecure attachment styles induce emotional disorders through affecting sleep. Sleep plays an important mediating role between attachment anxiety and depression, so we should pay attention to sleep quality and its related factors such as attachment and reappraisal, and methods should be taken to help create a supportive environment and improve students' sleep quality. In addition, our study suggested that poor sleep quality moderates insecure attachment with depression. Many previous studies have reported about insecure attachment with depression [38–39]; however, no previous study reported about the mediation role of poor sleep in the process. Poor sleep and lower level of cognitive reappraisal play a moderate role from insecure attachment to depression.

Cognitive reappraisal is a kind of emotional regulation that helps us to keep calm at stressful events. Emotion starts at the individual's perceiving a stimulus with a context and attending to its features. Every stimulus has two features: whether it happens as expected and whether it fits in our needs [40]. The individual appraises these two features of stimulus and triggers an affective, physiological, and behavioral response. The cognitive appraisal is a kind of emotional regulation strategy that helps the individual changing his interpretations or appraisal of the stimulus. The reason for its popularity in emotional regulation is that it is highly effective at regulating affect and physiological arousal without any physiological costs, and it has longer lasting effects than attention-focused strategies [41]. Thus, many studies have begun applying insights from behavioral and brain imaging research on reappraisal [42]. Here, our data showed that cognitive reappraisal might work as a buffer to help good sleep at stressful life events. Finally, as long as cognitive reappraisal acts as a buffer between attachment-related anxiety and depression as well as sleep quality, it is feasible to solve emotional problems by improving reappraisal level [43].

## 5. Limitation and Future Research Directions

Although the results of this study are interesting and will shed some light on the depression and sleep problems in college students, the results should be assessed with care, and the conclusions should be limited. First, our studies only provide a relationship study; it is hard to see the causal relationship. Second, our data were collected at only one single point; we are expecting a longitudinal study. Third, our study depends on self-reported questionnaire; we hope to have more reliable way to collect data or use animal studies in the future. In addition, neurobiological measurement can be detected in the brain, particularly in areas related to sleep quality and emotional disorders, including control of stress and emotions [44].

In conclusion, our study clarifies the relationship among insecure attachment and depression, through sleep problems. Previous studies about *monoamine theory for basic emotions* suggested that the monoamine neurotransmitters, including norepinephrine (NE), serotonin (5-HT), and dopamine (DA), as well as the cortisol system might be the major reason for emotional arousal and sleep disorders [45]. In our previous papers, we also proposed that the dopamine and norepinephrine are neural substrates for emotional arousal, which is the reverse of sleep [46]. Here, we suggested that sleep plays an important role in insecure attachment-induced emotional disorders, which might suggest that insecure attachment might also relate to the monoamines. In addition, we also found that sleep partially mediated the relationship between insecure attachment and depression. In all, we can make a conclusion that sleep quality can be predicted by insecure attachment directly, and sleep quality in turn can predict the depressive emotional problems. Our findings suggest that sleep quality and cognitive reappraisal play very important roles in depressive emotions.

## Figures and Tables

**Table 1:** Means, standard Deviations, and correlations for all variables (*N* = 755).

	*M*	*SD*	1	2	3	4	5
(1) Avoidance	2.82	0.84	—				
(2) Anxiety	3.68	1.05	0.29^∗∗^	—			
(3) Depression	3.83	0.59	0.16^∗∗^	0.33^∗^	—		
(4) Cognitive reappraisal	5.22	0.99	-0.17^∗∗^	0.01	0.04	—	
(5) Sleep quality	6.37	2.72	0.16^∗∗^	0.23^∗∗^	0.27^∗∗^	0.02	—

Note: ^∗∗^*p* < 0.01.

**Table 2:** Multiple regression analyses for the indirect effect of sleep.

Regression		Fix index			Significance of regression coefficient			
Dependent variable	Independent variable	*R*	*R*^2^	*F*	*β*	*LLCI*	*ULCI*	*t*
Sleep quality	Avoidance	0.24	0.06	23.76^∗∗∗^	0.32	0.69	5.28^∗∗∗^	2.68^∗∗^
	Anxiety				0.51	0.32	0.69	5.28^∗∗∗^

Depression	Avoidance	0.36	0.14	61.34^∗∗∗^	0.12	0.23	7.78^∗∗∗^	2.79^∗^
	Anxiety				0.22	0.18	0.27	9.94^∗∗∗^

Reappraisal	Avoidance	0.31	0.09	26.25^∗∗∗^	0.27	0.04	0.50	2.29^∗^
	Anxiety				0.32	0.12	0.52	3.21^∗∗^

Sleep quality	Depression				0.64	0.63	1.04	4.92^∗∗∗^

Note: *n* = 755. ^∗^*p* < 0.05, ^∗∗^*p* < 0.01, ^∗∗∗^*p* < 0.001. Continuous variables in the regression equation have been normalized. *LLCI* and *ULCI* represent the lower and the upper limitations of 95% bootstrap confidence interval, respectively.

**Table 3:** Multiple regression analyses of the moderate effect of cognitive reappraisal.

Regression		Fit index			Significance of regression coefficient			
Dependent variable	Independent variable	*R*	*R* ^2^	*F*	*β*	*LLCI*	*ULCI*	*t*
Depression	Avoidance	0.45	0.18	38.69^∗∗∗^	0.08	0.09	0.17	2.64^∗^
	Anxiety				0.23	0.18	0.28	10.82^∗∗∗^
	**Reappraisal**				-0.04	-0.01	0.08	1.62
	Anxiety × **reappraisal**				-0.08	0.04	0.12	3.81^∗∗∗^

Sleep quality	Avoidance	0.31	0.10	16.14^∗∗∗^	0.28	0.05	0.52	2.35^∗^
	Anxiety				0.31	0.11	0.51	3.08^∗∗^
	Depression				0.85	0.58	1.67	7.83^∗∗∗^
	**Reappraisal**				-0.07	-0.13	0.26	0.67
	Anxiety × **reappraisal**				-0.11	-0.06	0.29	1.26

Note: *n* = 755. ^∗^*p* < 0.05, ^∗∗^*p* < 0.01, ^∗∗∗^*p* < 0.001. Continuous variables in the regression equation have been normalized. LLCI and ULCI represent the lower and the upper limitations of 95% bootstrap confidence interval, respectively.

**Table 4:** The indirect effects at different levels of cognitive reappraisal.

Cognitive reappraisal	Indirect effect	*LLCI*	*ULCI*
*M*–*SD*	0.31	0.25	0.36
*M*	0.22	0.18	0.27
*M* + *SD*	0.14	0.08	0.21

Note: *LLCI* and *ULCI* represent the lower and the upper limitations of 95% bootstrap confidence interval, respectively.

## Data Availability

We will be very glad to share all the data underlying the findings of our manuscripts, in order to verify the results of an article, replicate the analysis, and conduct secondary analyses.
